# Introduction to special issue on “Nanoparticles in Medicine: Targeting, Optimization and Clinical Applications”

**DOI:** 10.1002/btm2.10012

**Published:** 2016-06-30

**Authors:** Paolo Decuzzi, Samir Mitragotri

**Affiliations:** ^1^ Laboratory of Nanotechnology for Precision Medicine, Italian Institute of Technology; ^2^ University of California

The precise delivery of therapeutic molecules and imaging agents to diseased tissues via rationally designed nanoparticles has offered a great promise and a challenge at the same time. Since the launch in 2005 of the Cancer Nanotechnology initiative from the National Cancer Institute, an incredibly large number of scientific manuscripts have been published on the use of nanoparticles for drug delivery and imaging. A quick PubMed search on “cancer” and “nanoparticles” would return over 15,000 articles published over the past two decades. Even more remarkable is the number of nanoformulated drugs that have entered the clinical trials: about 40 “nanomedicines” are currently under screening at different levels of clinical trials and several more are in the pipeline, and it is hoped that some of these will reach the clinic within the next few years. Given that the development of new therapies by the pharmaceutical industry takes between 10 and 15 years, the number of nanoparticles currently under clinical trials is remarkable and speaks for the strong efforts by the academic and industrial community to translate nanoparticles into clinical practice.

It is now well accepted that, as compared to individual molecules, nanoparticles have the ability to accumulate more avidly within the tumor tissues (*precision delivery*); release encapsulated cargo in a controlled fashion with the possibility of delivering multiple and different therapeutic molecules enabling combination therapies (*precision drugs*); and localize both imaging and therapeutic agents within the same tissue for simultaneous monitoring and delivery (*theranostics*). Systemically injected nanoparticles have been shown to reach tumors at concentrations as high as 10% of the injected dose per gram tissue in preclinical models depending on their design features, and this percentage can be two to three orders of magnitude higher than the tumor accumulation of conventional molecular‐based therapies. Literature has also revealed that such enhancements in accumulation are not to be taken for granted and substantial engineering of nanoparticles is essential to accomplish this goal. It is becoming increasingly common to use nanoparticles for simultaneously delivering multiple molecules for targeting both the tumor cells (chemotherapeutic agents) and the tumor microenvironment (immune cells, stroma, and others) with the objective of demolishing the cross‐talk between cancer and neighboring cells. Finally, nanoparticles for multimodal imaging, combining Positron Emitting Tomography (PET), Computer Tomography (CT), Magnetic Resonance (MR), and optical imaging with drugs, are commonly produced in several laboratories. Together with patient‐specific omics analyses*, precision delivery*; *precision drugs;* and *theranostics* represent the pillars of the medicine for the future: *precision medicine*.

Following this vision, this special issue focuses on different strategies that have been proposed to enhance the efficacy of nanoparticles in the treatment of oncological and cardiovascular diseases. This includes the use of molecular targeting and the rational design of geometrical and physical attributes of nanoparticles—size, shape, and elasticity—for enhancing the recognition of target cells and tissues; the realization of sophisticated *in vitro* microfluidic devices for testing the therapeutic efficacy of nanoparticles under a variety of controlled biophysical conditions; and the optimization of nanoparticle synthesis for enabling scaling up and clinical testing. Seven manuscripts are collected in this special issue. Eniola‐Adefeso and colleagues^1^ report on the advantages of using multiple targeting moieties for enhancing the vascular accumulation of nanoparticles; Toy and Roy^2^ review the work done on engineering the nanoparticle geometrical and mechanical properties for immunotherapy; and Zhang and collaborators^3^ summarize findings on using biomimetic strategies to modulate nanoparticle circulation and tumor accumulation. In a different set of manuscripts, Gupta and colleagues^4^ discuss microfluidics‐based *in vitro* assays for optimizing the performance of nanoparticles; and Chrzanowski and coworkers^5^ present strategies to maximize the cytotoxic effect of gold nanoparticles on colon cancer. Finally, Lahann and coworkers^6^ focus on electrospraying techniques for the synthesis of nanoparticles with precise geometry control; and Mitragotri and Anselmo^7^ review the current clinical status of nanomedicines.

Efforts to date from the academic and industrial community toward the development of nanoparticle‐based therapies are undoubtedly of great proportions. These efforts have brought substantial advances in scientific understanding, but at the same time, they have revealed numerous translational challenges. Future efforts must focus on addressing these challenges so as to facilitate the translation of nanoparticles into human applications.


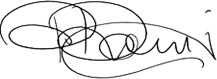


Paolo Decuzzi Senior Researcher, Professor, and Director *Laboratory of Nanotechnology for Precision Medicine Italian Institute of Technology Via Morego*,
*30 16163 Genova, ITALY*
Email: Paolo.Decuzzi@iit.it

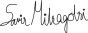


Samir Mitragotri *Deparment of Chemical Engineering Center for Bioengineering University of California Santa Barbara*,
*CA 93106*
Email: samir@engr.ucsb.edu
